# Asparagus Polysaccharide Suppresses the Migration, Invasion, and Angiogenesis of Hepatocellular Carcinoma Cells Partly by Targeting the HIF-1*α*/VEGF Signalling Pathway In Vitro

**DOI:** 10.1155/2019/3769879

**Published:** 2019-05-26

**Authors:** Wei Cheng, Ziwei Cheng, Dongwei Xing, Minguang Zhang

**Affiliations:** Shanghai Municipal Hospital of Traditional Chinese Medicine, Shanghai University of Traditional Chinese Medicine, Shanghai 200071, China

## Abstract

Hypoxia-inducible factor-1*α* (HIF-1*α*) plays a key role by triggering the transcriptional activation of a number of genes involved in migration, invasion, and angiogenesis in hepatocellular carcinoma (HCC). Thus, suppressing tumour growth by targeting the HIF-1*α*/VEGF signalling pathway represents a promising strategy for the treatment of HCC. In our previous studies, we found that asparagus polysaccharide (ASP) suppressed the proliferation and promoted the apoptosis of HCC cells both in vivo and in vitro. To further explore the potential mechanisms of the antitumor effects of ASP in HCC, we investigated effects of ASP on the migration, invasion, and angiogenesis of HCC cells (SK-Hep1 and Hep-3B) using an in vitro experimental model. First, we found that ASP effectively suppressed the proliferation of the SK-Hep1 and Hep-3B cells but did not cause significant cytotoxicity in normal liver cells (L-O2). Then, we found that ASP inhibited the migration and invasion of the SK-Hep1 and Hep-3B cells and HCC cells-induced angiogenesis of human umbilical vein endothelial cells in a concentration-dependent manner. Mechanistic studies revealed that the inhibition of migration, invasion, and angiogenesis by ASP in the SK-Hep1 and Hep-3B cells might occur via the downregulation of HIF-1*α*/VEGF signalling pathway. Finally, our results also showed that the inhibition of HIF-1*α* by ASP may be mediated through the downregulation of the phosphorylation levels of AKT, mTOR, and ERK. In conclusion, our results suggest that ASP suppresses the migration, invasion, and angiogenesis of HCC cells partly via inhibiting the HIF-1*α*/VEGF signalling pathway.

## 1. Introduction

Hepatocellular carcinoma (HCC) has become the fifth common tumour and the second major cause of tumour-related death worldwide [1, 2]. Half of these new cases and deaths occur in China [3]. Liver tumour resection and liver transplantation are the main treatments for early HCC, and these patients have relatively high 1-, 3-, and 5-year overall survival rates [4]. However, most patients with HCC are in advanced stage or local advanced stage when diagnosed, at which point surgery or liver transplantation is not a treatment option. Transcatheter arterial chemoembolisation (TACE), radiotherapy, chemotherapy, and sorafenib-targeted therapy are the main treatments for advanced HCC [5]. However, those therapeutic strategies have relatively severe adverse effects. Thus, the identification of effective treatment strategies for HCC with no or few adverse effects is urgently needed.

Migration and invasion are important steps in the progression and recurrence of HCC and determine the success and failure of liver cancer treatment to some extent [6, 7]. Inhibition of migration and invasion in HCC is the current trend in liver cancer research [8, 9]. Angiogenesis also plays a vital role in the development of HCC, which is a highly vascularised solid tumour [10]. Vascular endothelial growth factor (VEGF) is an efficient inducer of the migration, invasion, and angiogenesis of HCC cells [11–15]. Thus, VEGF blocking therapy is an important approach for the treatment of HCC [16, 17]. Bevacizumab, the most commonly used antiangiogenic drug, can independently inhibit HCC progression by downregulating the VEGF/VEGFR pathway [18]. However, these drugs always increase tumour hypoxia and lead to a poor prognosis [19].

Hypoxia-inducible factor-1 (HIF-1) is a heterodimer protein containing an oxygen-sensitive *α* subunit and a constantly expressed *β* subunit [20, 21]. HIF-1*α* plays an important role in promoting the migration, invasion, and angiogenesis of HCC by activating the transcription of human VEGF genes and encoding VEGF protein [22–24]. Although the expression of HIF-1*α* is relatively low in normoxia compared with that in hypoxia, it is still highly expressed in some malignant tumour cell lines. Several studies have reported that traditional Chinese medicine can inhibit tumour cells growth and angiogenesis by suppressing the expression of HIF-1*α* in normoxia [25–27].

Asparagus, a common Chinese herb, is often applied for the treatment of malignant tumours and chronic inflammation [28–30]. ASP, an extract from asparagus, is a type of polysaccharide which has been shown to be the main active ingredient of asparagus involved in its antitumor and apoptosis-promoting activities. In our previous studies, we reported that ASP has relatively few side effects compared with common antitumor drugs and can inhibit tumour cell proliferation, promote tumour cell apoptosis, and prolong the survival of tumour-bearing animals [31–35]. The effects of ASP on the growth, migration, and invasion of Hep-G2 cells under hypoxic conditions have also been reported by members of our research group [36]. However, there have been no published studies to date on the effects of ASP on migration, invasion, and angiogenesis in the Sk-Hep1 and Hep-3B cells. Thus, to better understand the potential mechanism of the antitumor activity of ASP, the inhibition of migration and invasion by ASP and its antiangiogenic effects in vitro was investigated in human HCC cells lines (Sk-Hep1 and Hep-3B).

## 2. Materials and Methods

### 2.1. Materials and Reagents

Dulbecco's modified Eagle's medium (DMEM) and penicillin/streptomycin were purchased from Hyclone (Logan, USA). Foetal bovine serum (FBS) was purchased from Gibico (South America). HIF-1*α* antibody was purchased from Abcam (Cambridge, United Kingdom). VEGF antibody was purchased from RD (Minneapolis, USA). Antibodies against AKT, p-AKT, mTOR, p-mTOR, ERK, p-ERK, MMP2, and MMP9 were purchased from CST (Boston, USA). Horseradish peroxidase- (HRP-) conjugated secondary antibodies, fluorescent-conjugated secondary antibodies, New Super ECL Assay, and *β*-actin antibody were purchased from KeyGEN BioTECH (Nanjing, China). Transwell chambers and matrigel were purchased from Corning Life Sciences (8 *μ*m pores, Tewksbury, MA, USA) and BD Biosciences (10.5mg/ml, San Jose, CA, USA), respectively. The QRT-PCR Kits were purchased from Takara (Shiga, Japan). The Cell Counting kit-8 (CCK-8) was purchased from Dojindo Molecular Technologies, Inc. (Kumamoto, Japan). The enzyme-linked immune sorbent assay (ELISA) kits for human VEGF were purchased from Multi Sciences (Shanghai, China). BCA Protein Assay Kits were purchased from Beyotime Biotechnology.

### 2.2. Cell Culture

Human umbilical vein endothelial cells (HUVECs) line and the L-O2, SK-Hep1, and Hep-3B cells lines were purchased from the Type Culture Collection of the Chinese Academy of Sciences (Shanghai, China) and then cultured in 5% CO_2_ at 37°C in DMEM with 10% FBS and 1% penicillin-streptomycin.

### 2.3. Obtainment of ASP

ASP was purchased from Shanghai Yuanye Bio-Technology Co, Ltd (Shanghai, China). DMEM was used to dissolve ASP to a final concentration of 200 mg/ml. Then, the supernatant was obtained by centrifugation at 200×g for 5 min and stored at -20°C for long-term use.

### 2.4. CCK-8 Cell Viability Assay

CCK-8 was used to assess cell viability. First, 1×10^4^ cells/well were plated onto 96-well culture plates and then cultured for 24 h prior to treatment. ASP was dispensed into the 96-well culture plates at a final concentration of 30, 20, 10, 5, 2.5, 1.25, and 0 mg/ml, and cells were incubated for another 24 h. The cells were washed three times with sterile PBS, and, then, 10 *μ*l CCK-8 and 90 *μ*l fresh medium were added. Finally, cells were incubated for 1 h at 37°C. The optical density (OD) at 450 nm was measured using a microplate reader. All measurements were carried out in triplicate. The relative inhibition rate of cell viability was calculated according to the formula R = [(A2-A1)/A2] ×100%. R is the relative inhibition rate, A1 is the mean absorbance value of cells treated with ASP, and A2 is the mean absorbance value of cells without any drug treatment.

### 2.5. Wound Healing Assay

Cells were plated in six-well plates. A straight line was traced out in the middle of each well with a white sterile pipette tip on the cell layer after it had reached 80-90% confluence. Then, the cells were rinsed three times with fresh serum-free DMEM to wash away cells debris. Next, 2 ml complete medium containing various concentrations of ASP (0, 2.5, 5, and 10 mg/ml) was added to these cells followed by incubation for 24 h. Scratched cells were photographed with an inverted microscope at 0 h and 24 h. These experiments were repeated three times.

### 2.6. Transwell Invasion Assay

We used a transwell invasion assay to assess cell invasion. First, 600 *μ*l DMEM containing 20% FBS was dispensed into the lower chamber. Then, 100 *μ*l matrigel diluted with fresh serum-free DMEM at a 1:3 ratio was dispensed into the upper chamber and allowed to solidify for 30 min at 37°C before cells were plated. Then, 5×10^4^ HCC cells pretreated with various concentrations of ASP (0, 2.5, 5, and 10 mg/ml) for 24 h were plated into the upper chamber. The invasion assay plate was incubated for 72 h. After that, invasive cells were analysed by crystal violet staining, and the number of cells was counted under a light microscope. These experiments were repeated three times.

### 2.7. HUVECs Tube Formation Assay

HCC cells were treated with various concentrations of ASP (0, 2.5, 5, and 10 mg/ml) for 24 h, and, then, the cell supernatants were obtained as conditioned medium for subsequent use. For the assay, 50 *μ*l matrigel was dispensed into 96-well plates and allowed to solidify at 37°C for 30 min, and 100 *μ*l conditioned medium was added to the 96-well plates with matrigel. HUVECs (2.5×10^4^ cells) were seeded on the matrigel and incubated at 37°C for 24 h. The formation of capillary-like structures was photographed, and the tube number was analysed using Image J. These experiments were repeated three times.

### 2.8. ELISA Assay

HCC cells were treated with various concentrations of ASP (0, 2.5, 5, and 10 mg/ml) for 24 h. Then, the cell supernatants were obtained. We used ELISA kit to detect the level of secreted VEGF in the culture supernatant of HCC cells according to the manufacturer's protocol. The concentration of VEGF was expressed as the fold change relative to that of cells in the control group (0 mg/ml ASP).

### 2.9. Quantitative RT-PCR Assay

Total RNA of HCC cells treated with various concentrations of ASP (0, 2.5, 5, and 10 mg/ml) for 24 h was extracted using TRIzol Reagent according to the manufacturer's instructions. QRT-PCR kit and 0.5 *μ*g total RNA were used to carry out the qRT-PCR assay. All of the primers were synthesised by Sangon Biotech. The specific primers are listed in Table 1.

### 2.10. Western Blotting Assay

HCC cells treated with various concentrations of ASP (0, 2.5, 5, and 10 mg/ml) for 24 h were scraped off six-well culture plates and lysed with RIPA lysis buffer containing protease inhibitors and phenylmethylsulfonyl fluoride (PMSF) for 30 min on ice. Protein concentration was determined with BCA Assay Kit. Equal amounts of protein (50-100 *μ*g) and the corresponding 5×loading buffer were mixed, subjected to electrophoresis on 7.5% or 12% sodium dodecyl sulfate-polyacrylamide gels, and then transferred to a Pure Nitrocellulose Blotting Membrane. After they were blocked with 5% non-fat milk at room temperature for 2 h, the membranes were incubated overnight at 4°C with primary antibodies (diluted at 1:1000), followed by incubation with HRP-conjugated secondary antibodies (diluted at 1:2000) at room temperature for 1 h. Targeted proteins were visualised using a New Super ECL Assay and then exposed to film.

### 2.11. Immunofluorescence

HCC cells grown on coverslips were treated with various concentrations of ASP (0, 2.5, 5, and 10 mg/ml) for 24 h, then fixed in 4% paraformaldehyde (PFA) for 15 min, and blocked with blocking buffer containing 3% FBS, 1% goat serum, and 0.1% Triton X-100 for 2 h at room temperature. Then, the cells were incubated overnight at 4°C with the primary antibody (diluted at 1:200) followed by incubation with fluorescent-conjugated secondary antibodies (diluted at 1:1000) for 1 h at room temperature. Subsequently, cells were incubated with DAPI for 10 min at room temperature. Finally, cells were observed and photographed under a fluorescence microscope.

### 2.12. Statistical Analysis

All data were presented as the mean ± standard deviation, and the significance of the difference between groups was estimated by two-tailed analysis of variance (ANOVA). Statistical analyses were carried out using IBM SPSS Statistics 21.* P*<0.05 was considered significant for all tests.

## 3. Results

### 3.1. ASP Inhibited the Proliferation of HCC Cells

**Figure 1:**
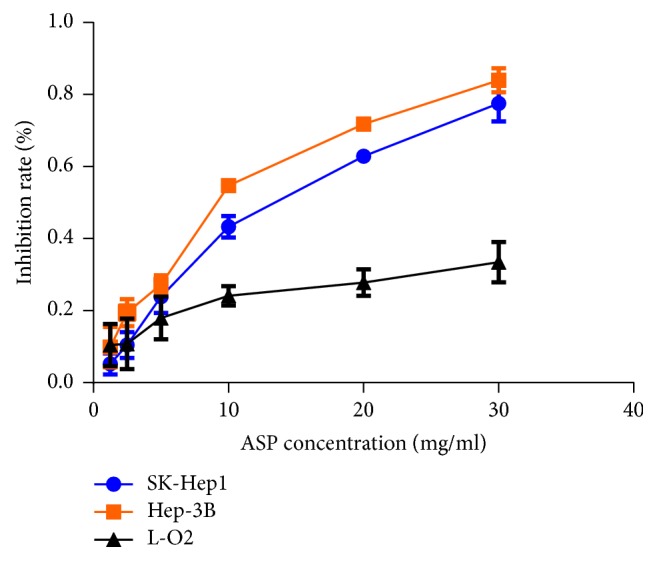
Inhibition of cell proliferation was measured using CCK-8 assay in SK-Hep1, Hep-3B, and L-02 cells treated with ASP at the concentrations of 30, 20, 10, 5, 2.5, 1.25, and 0 mg/ml for 24 h. Data were graphed using GraphPad Prism 5.02.

To evaluate anticancer effect of ASP, we used different concentrations of ASP (30, 20, 10, 5, 2.5, 1.25, and 0 mg/ml) to treat HCC cells (SK-Hep1 and Hep-3B) and normal liver cells (L-02) for 24 h. The results indicated that ASP inhibited the proliferation of HCC cells in a concentration-dependent manner (Figure 1). Then, 10 mg/ml was chosen as the high concentration of ASP for subsequent experiments based on the IC50 value (12.81 mg/ml for SK-Hep1 and 9.04 mg/ml for Hep-3B). In addition, it was found that ASP has no significant cytotoxicity to L-02 cells (Figure 1).

### 3.2. ASP Inhibited the Migration and Invasion of HCC Cells

**Figure 2:**
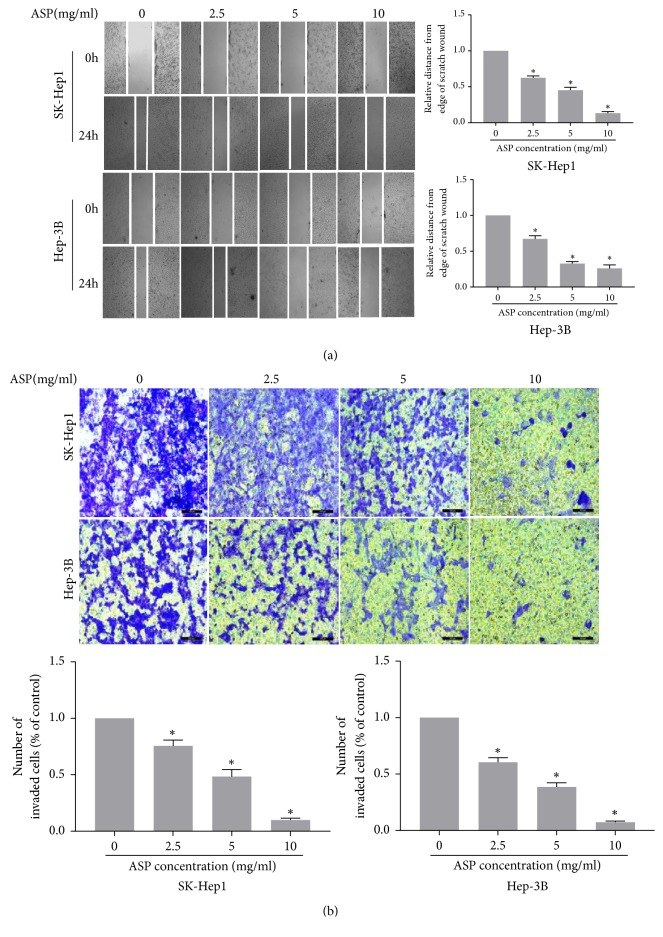
ASP inhibited the migration and invasion of HCC cells. (a) Wound healing assay was performed to test the effect of ASP (0, 2.5, 5, and 10 mg/ml, for 24 h) on migration in SK-Hep1 and Hep-3B cells. The same point of view was selected to take photographs at 0 h and 24 h. Histogram presented the migration distance relative to that of the control group for 24 h after scratching. Data were graphed using GraphPad Prism 5.02. *∗*,* p*<0.05 compared with the control group. (b) Transwell invasion assay was performed to test the effect of ASP (0, 2.5, 5, and 10 mg/ml, for 72 h) on invasion in SK-Hep1 and Hep-3B cells. Cells on the lower surface of the transwell membrane were stained with 0.1% crystal violet. Histogram presented the average number of invaded cells per field. Data were graphed using GraphPad Prism 5.02. *∗*,* p *< 0.05 compared with the control group.

**Figure 3:**
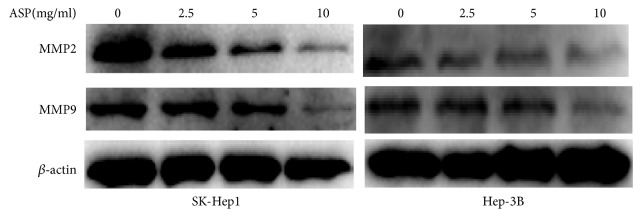
Protein expression of MMP2 and MMP9 in SK-Hep1 and Hep-3B cells treated with ASP (0, 2.5, 5, and 10 mg/ml, for 24 h).

To assess the inhibitory effect of ASP on migration and invasion, we used different concentrations of ASP (0, 2.5, 5, and 10 mg/ml) to treat HCC cells (SK-Hep1 and Hep-3B) for 24 h (migration) or 72 h (invasion). The results indicated that ASP obviously suppressed the migration and invasion of HCC cells in a concentration-dependent manner (Figure 2). The western blotting results indicated that ASP inhibited the expression of MMP2 and MMP9 (Figure 3).

### 3.3. ASP Inhibited HCC Cells-Induced Tube Formation of HUVECs

**Figure 4:**
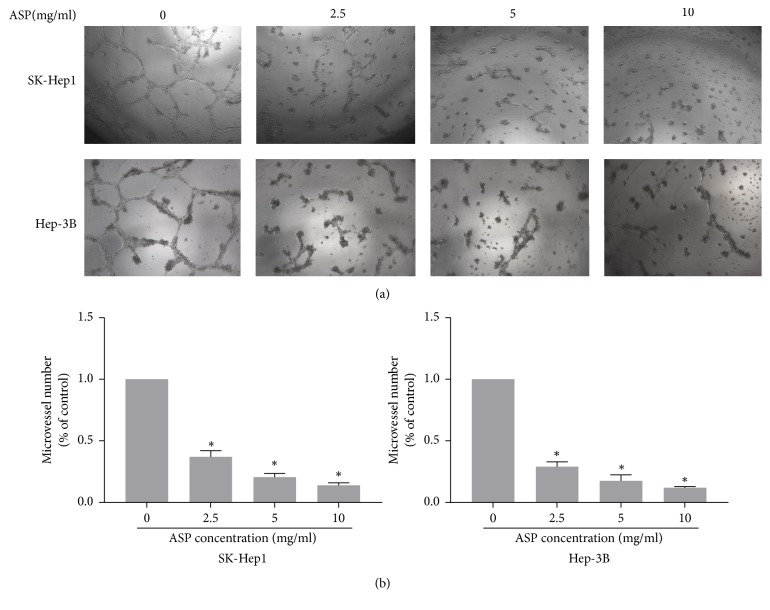
Effect of ASP on HCC cells-induced tube formation of HUVECs. (a) The medium from SK-Hep1 and Hep-3B cells treated previously with ASP (0, 2.5, 5, and 10 mg/ml, for 24 h) was collected and then applied to tube formation assay. (b) Representative histogram of the area covered by the tube network quantitated using Image-Pro Plus software. *∗*,* p* < 0.05, compared with the control group.

To assay the effect of ASP on the HCC cells-induced tube formation of HUVECs, we performed a tube formation assay. Our results indicated that completed network structures were significantly decreased by the different concentrations of ASP (2.5, 5, and 10 mg/ml) compared with that in the control group (0 mg/ml ASP) in a concentration-dependent manner (Figure 4).

### 3.4. ASP Inhibited the Expression of HIF1*α* and VEGF in HCC Cells

**Figure 5:**
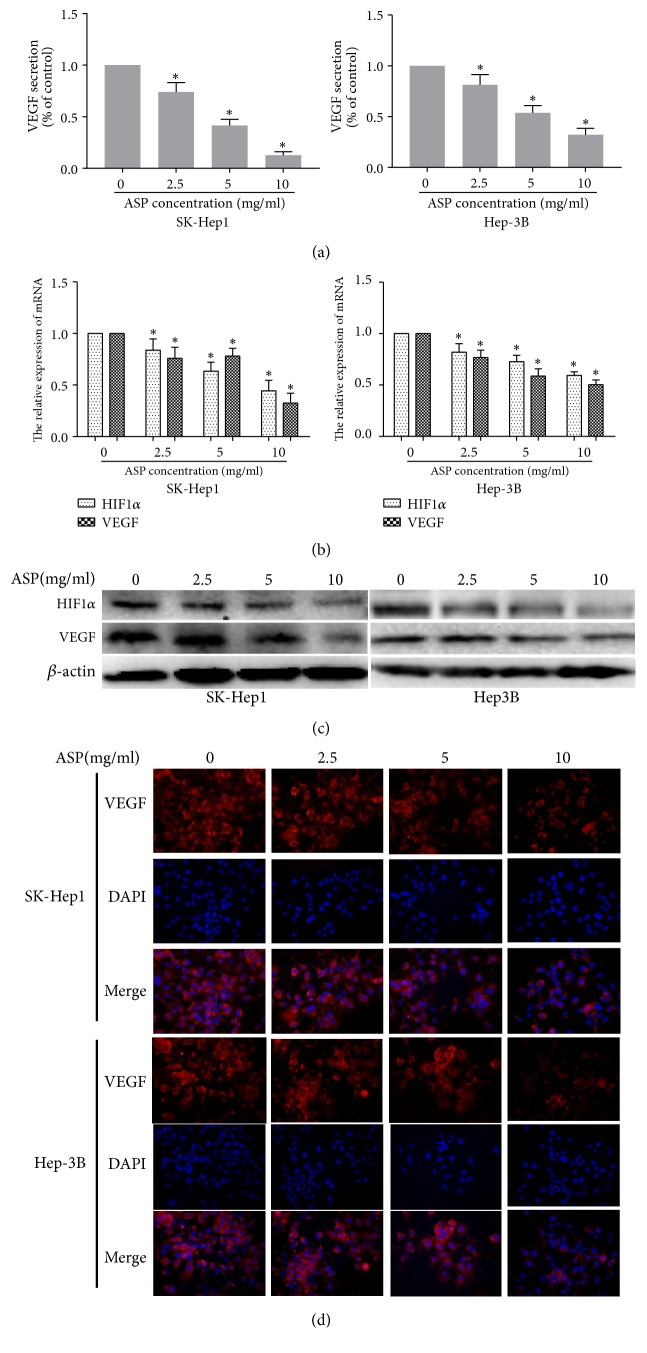
Effects of ASP (0, 2.5, 5, and 10 mg/ml, for 24 h) on the expression of HIF-1*α* and VEGF in SK-Hep1 and Hep-3B cells. (a) Secreted VEGF protein was analysed by ELISA. (b) mRNA expression levels of HIF-1*α* and VEGF were analysed by qRT-PCR. (c) Protein expression levels of HIF-1*α* and VEGF were analysed by western blotting, and *β*-actin was used as a loading control. (d) Protein expression levels of VEGF were analysed by immunofluorescence. *∗*,* p* < 0.05 compared with the control group.

To further determine the mechanism by which ASP inhibits the migration, invasion, and angiogenesis of HCC cells, we assayed the expression of HIF1*α* and VEGF in HCC cells using ELISA, qRT-PCR, western blotting, and immunofluorescence. We used ELISA kit to detect the effect of ASP on VEGF secretion in HCC cells (SK-Hep1 and Hep-3B) and found that the different concentrations of ASP (2.5, 5, and 10 mg/ml) significantly suppressed VEGF secretion by HCC cells in a concentration-dependent manner (Figure 5(a)). Then, our qRT-PCR assay revealed that the different concentrations of ASP (2.5, 5, and 10 mg/ml) significantly inhibited the mRNA expression of HIF-1*α* and VEGF in SK-Hep1 and Hep-3B cells compared with that in the control group (0 mg/ml ASP) (Figure 5(b)). The western blotting assay further indicated that the different concentrations of ASP (2.5, 5, and 10 mg/ml) significantly decreased the protein expression of HIF-1*α* and VEGF in SK-Hep1 and Hep-3B cells (Figure 5(c)). Additionally, immunofluorescence assay also revealed that the addition of ASP obviously reduced the expression of VEGF in SK-Hep1 and Hep-3B cells (Figure 5(d)).

### 3.5. Inhibitory Effect of ASP on HIF-1*α* Expression Was Mediated by Blocking the Phosphorylation of MAPK and PI3K Signalling Pathways in HCC Cells

**Figure 6:**
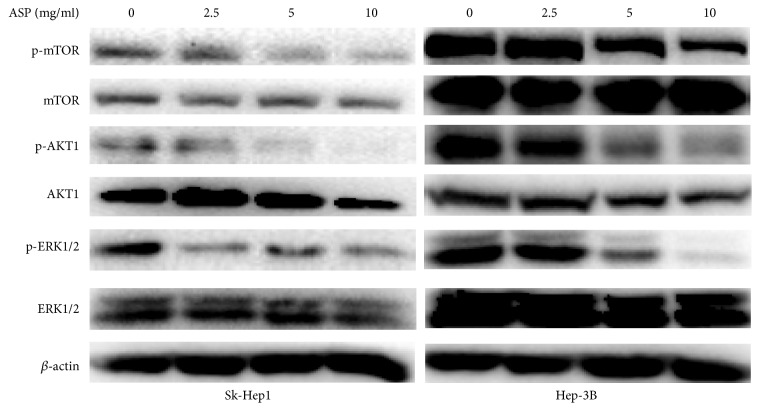
Effects of ASP (0, 2.5, 5, and 10 mg/ml, for 24 h) on the protein levels of AKT, p-AKT, mTOR, p-mTOR, ERK, and p-ERK in SK-Hep1 and Hep-3B cells analysed by western blotting; *β*-actin was used as a loading control.

It is commonly known that the MAPK and PI3K signalling pathways play a pivotal role in the regulation of HIF1*α* protein expression [37, 38]. To determine whether the inhibition of HIF-1*α* protein expression by ASP was mediated by the MAPK and PI3K signalling pathways, we measured the protein levels of AKT, p-AKT, mTOR, p-mTOR, ERK, and p-ERK after ASP (0, 2.5, 5, and 10 mg/ml) treatment for 24 h in SK-Hep1 and Hep-3B cells. As shown in Figure 6, the protein levels of p-AKT, p-mTOR, and p-ERK in the SK-Hep1 and Hep-3B cells were decreased by the different concentrations of APS, while no significant changes in the protein levels of AKT, mTOR, and ERK were found. These results indicated that the inhibition of HIF1*α* expression by ASP might be achieved through downregulating the phosphorylation levels of AKT, mTOR, and ERK.

## 4. Discussion

HCC is currently recognised as a refractory malignancy worldwide, and its morbidity and mortality have increased significantly, especially in China, in recent years. Although there are many treatment strategies for HCC, most of the treatments are associated with severe adverse reactions, and the treatment effect remains unsatisfactory. The overall survival rate of patients with HCC is still low [39]. Therefore, there is an urgent need for a drug or treatment with few adverse reactions and definite therapeutic effect to overcome the shortcomings of current HCC treatments.

Migration plays a key role in the prognosis of malignant tumours, and most HCC-related deaths are attributed to migration occurring [40]. Tumour invasion is one of the basic biological characteristics of malignant tumours, and it is also one of the important causes of death in most patients with tumours [41]. HCC is a highly vascular tumour, and angiogenesis plays a critical role in the progression of HCC. The proliferation, migration, and invasion of HCC require the development of new blood vessels, and angiogenesis must be inhibited to control the growth of HCC [42]. Thus, the inhibition of tumour migration, invasion, and angiogenesis is an important research direction for the development of new tumour treatments.

HIF-1*α* has been reported to be significantly upregulated in HCC tissues compared with that in the normal liver tissue, and the overexpression of HIF-1*α* was associated with a short overall survival and disease-free survival in patients with HCC [43, 44]. Additionally, HIF-1*α* has also been shown to drive VEGF expression and then promote migration, invasion, and angiogenesis [45]. Moreover, many studies have reported that drugs targeting the HIF-1*α*/VEGF signalling pathway suppressed the growth of liver tumours and represented a promising therapeutic strategy for patients with HCC in the future [46–50].

At present, Chinese medicine has achieved remarkable achievements in the treatment of HCC, and the associated research results are increasingly recognised by scholars in China and abroad [51–53]. Previous studies have demonstrated that ASP can significantly inhibit the proliferation and apoptosis and prolong the survival of liver cancer-bearing mice. Additionally, the suppressive effects of ASP on the proliferation, migration, and invasion of the Hep-G2 cells under hypoxic conditions were also reported [36]. However, the effects of ASP on the migration, invasion, and angiogenesis of Sk-Hep1 and Hep-3B cells were unclear. Thus, we performed the current study to explore the effects of ASP on the proliferation, migration, invasion, and angiogenesis of HCC cells (SK-Hep1 and Hep-3B), as well as the associated mechanisms and signalling pathways.

Our results indicated that ASP inhibited the proliferation, migration, and invasion of SK-Hep1 and Hep-3B cells as well as HCC cells-induced angiogenesis in a concentration-dependent manner. Mechanistic studies revealed that ASP significantly inhibited the expression of HIF-1*α* and VEGF at both the protein and mRNA levels in SK-Hep1 and Hep-3B cells. Finally, our results further indicated that ASP suppressed p-AKT, p-mTOR, and p-ERK but not AKT, mTOR, and ERK in the SK-Hep1 and Hep3B cells. These results will provide an experimental basis for the late clinical application of ASP.

There were several limitations in our study. First, our results need to be verified in other HCC cells lines, such as the Huh7, bel7404, and SMMC-7721 cells lines. Second, the inhibitory effects of ASP on HIF-1*α*, p-mTOR, p-AKT1, and p-ERK in HCC cells need to be verified using signalling pathway inhibitors. Finally, our results also should be verified with in vivo experiments.

**Figure 7:**
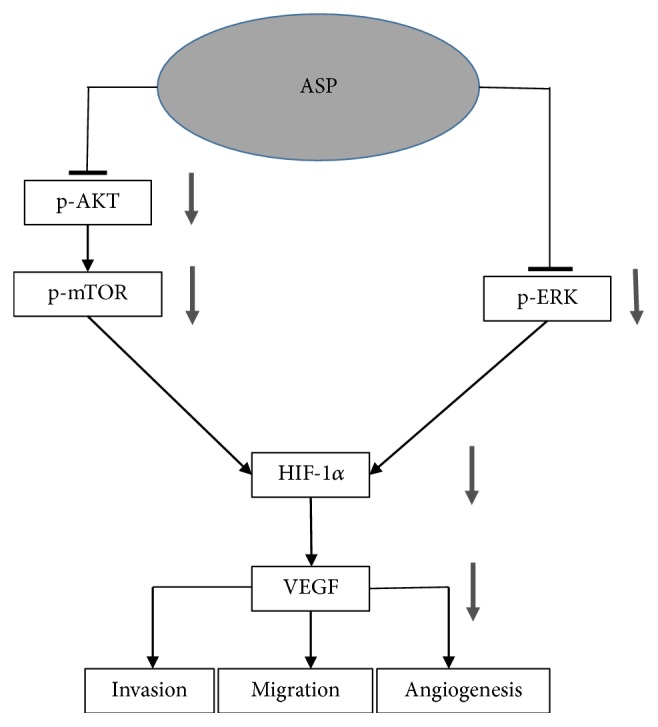
Proposed mechanism for the inhibitory effects of ASP on the migration, invasion, and angiogenesis of HCC cells. Connector lines represent established pathways. Arrows indicate regulation by ASP treatment based on the experimental results. ASP, asparagus polysaccharide; HIF-1*α*, hypoxia-inducible factor 1 alpha; VEGF, vascular endothelial growth factor.

In conclusion, our results suggest that ASP suppresses the migration, invasion, and angiogenesis of HCC cells partly through the downregulation of HIF-1*α*/VEGF expression via the PI3K and MAPK signalling pathways (Figure 7). ASP, an extract of a widely used traditional Chinese medicine with low toxicity to normal hepatocytes and potent antitumor activity, deserves further research to determine its possible applications in the therapy of liver cancer.

## Figures and Tables

**Table 1:** Primers used for qRT-PCR.

name	Sequences (5'-3')
GAPDH-F	CAGGAGGCATTGCTGATGAT
GAPDH-R	GAAGGCTGGGGCTCATTT
HIF-1*α*-F	TGACAAGCCACCTGAGGAGA
HIF-1*α*-R	ACACGCGGAGAAGAGAAGGA
VEGFA-F	TACCTCCACCATGCCAAGTG
VEGFA-R	GGTCTCGATTGGATGGCAGT

## Data Availability

The data used to support the findings of this study are included within the article.
